# Testing multi-theory model (MTM) in predicting initiation and sustenance of physical activity behavior among college students

**DOI:** 10.15171/hpp.2016.11

**Published:** 2016-06-11

**Authors:** Vinayak K. Nahar, Manoj Sharma, Hannah Priest Catalano, Melinda J. Ickes, Paul Johnson, M. Allison Ford

**Affiliations:** ^1^Department of Health, Physical Education, and Exercise Science, School of Allied Health Sciences, Lincoln Memorial University, Harrogate, TN, USA; ^2^Behavioral & Environmental Health, School of Public Health, Jackson State University, MS, USA; ^3^Public Health Studies, School of Health and Applied Human Sciences, University of North Carolina Wilmington, NC, USA; ^4^Department of Kinesiology and Health Promotion, College of Education, University of Kentucky, KY, USA; ^5^Department of Management, School of Business Administration, University of Mississippi, MS, USA; ^6^Department of Health, Exercise Science & Recreation Management, School of Applied Sciences, University of Mississippi, MS, USA

**Keywords:** College students, Physical activity, Multi-theory model, Exercise, Needs assessment

## Abstract

**Background:** Most college students do not adequately participate in enough physical activity (PA) to attain health benefits. A theory-based approach is critical in developing effective interventions to promote PA. The purpose of this study was to examine the utility of the newly proposed multi-theory model (MTM) of health behavior change in predicting initiation and sustenance of PA among college students.

**Methods:** Using a cross-sectional design, a valid and reliable survey was administered in October 2015 electronically to students enrolled at a large Southern US University. The internal consistency Cronbach alphas of the subscales were acceptable (0.65-0.92). Only those who did not engage in more than 150 minutes of moderate to vigorous intensity aerobic PA during the past week were included in this study.

**Results:** Of the 495 respondents, 190 met the inclusion criteria of which 141 completed the survey. The majority of participants were females (72.3%) and Caucasians (70.9%). Findings of the confirmatory factor analysis (CFA) confirmed construct validity of subscales (initiation model: χ2 = 253.92 [df = 143], P < 0.001, CFI = 0.91, RMSEA = 0.07, SRMR = 0.07; sustenance model: χ2= 19.40 [df = 22], P < 0.001, CFI = 1.00, RMSEA = 0.00, SRMR = 0.03). Multivariate regression analysis showed that 26% of the variance in the PA initiation was explained by advantages outweighing disadvantages, behavioral confidence, work status, and changes in physical environment. Additionally, 29.7% of the variance in PA sustenance was explained by emotional transformation, practice for change, and changes in social environment.

**Conclusion:** Based on this study’s findings, MTM appears to be a robust theoretical framework for predicting PA behavior change. Future research directions and development of suitable intervention strategies are discussed.

## Introduction

There are numerous long- and short-term benefits of physical activity (PA). Long-term benefits include reduced risk of overall morbidity, heart disease, hypertension, type 2 diabetes, metabolic syndrome, and some cancers.^1^ Both prevention of weight gain and promotion of weight loss are also linked to achieving recommended levels of PA.^1^ Additional benefits pertinent among college students include improved mental health, enhanced quality of sleep, and ability to deal with academic demands.^1^ Despite the importance of PA for health and obesity prevention, less than half (46%) of US college students meet recommendations for moderate-intensity exercise, vigorous-intensity exercise, or a combination of the two.^2^ In fact, almost one in four college students report zero days of moderate-intensity aerobic exercise for at least 30 minutes.^2^

There is a clear need to understand factors influencing PA during the college years, a time of transition to lifetime behaviors.^3^ College students have reported individual level (e.g., perceived benefits, perceived barriers, enjoyment), psychosocial level (e.g., social support, modeling, self-efficacy), and environmental level reasons (e.g., availability and accessibility) for participating in or deterring from participation in PA.^4-9^ Although a range of theoretical models have been used to identify such factors, the existing health behavior theories and models have conceptual problems,^10,11^ lack predictive power,^11-14^ are not parsimonious,^13,15^ and/or are too comprehensive,^16-18^ and consequently, impractical.

In recognition of these issues, Sharma recently proposed a multi-theory model (MTM) for health behavior change, using constructs that have been extensively validated with a broad range of populations in cross-cultural settings.^17^ The MTM is a parsimonious model that was exclusively developed for health education and health promotion to explain and predict one-time and long-term health behavior change, and may be applied at individual, group, and community levels.^17^ Sharma posits that health behavior change can be dichotomized into two components: (1) initiation of the health behavior change and (2) sustenance of the health behavior change.^17^ Initiation of the health behavior change involves transitioning from one behavior to a different one. Initiation of the health behavior change includes participation in a one-time behavior such as a single-dose vaccination. Sustenance of the health behavior change involves long-term performance of the behavior change, such as engaging in PA throughout the course of the lifetime. Further, Sharma indicates that this dissection is necessary because the constructs that affect initiation of health behavior differ from those that affect sustenance of health behavior.

The MTM poses that three primary constructs explain and predict the initiation of health behavior change. These include participatory dialogue, behavioral confidence, and changes in physical environment. Derived from Freire’s model of adult education, “participatory dialogue” is two-way communication that emphasizes the advantages and disadvantages of a health behavior change.^19^ “Participatory dialogue” is related to the perceived benefits and perceived barriers constructs in the health belief model (HBM),^11^ and pros and cons in the trans-theoretical model (TTM);^13^ however, it differs in the process, which emphasizes communication that is participatory, and can be initiated by the health educator. The “behavioral confidence” construct is developed from Ajzen’s perceived behavioral control,^20^ and Bandura’s self-efficacy constructs.^21^ “Behavioral confidence” differs from these constructs as its focus is on changing behavior and the source of confidence is not exclusive to self.^17^ For instance, behavioral confidence may come from external sources including significant individuals or groups in life, a health educator, God, etc.^17^ This construct attempts to measure how certain someone is to engage in a health behavior change in the future rather than the present.^17^ The “changes in physical environment” construct is developed from Prochaska’s environmental re-evaluation construct,^13^ Bandura’s environment construct,^21^ and Fishbein’s environmental factors within the integrative model.^22^ This construct is specific to the physical environment only and not the social environment. “Changes in physical environment” involves modifying the “obtainability, availability accessibility, convenience, and readiness of resources.”^17^

Furthermore, the MTM includes three additional constructs which influence sustenance of health behavior change; these constructs include emotional transformation, practice for change, and change in social environment. The “emotional transformation” construct is derived from the self-motivation construct of the emotional intelligence theory.^23^ “Emotional transformation” involves altering emotions and directing them to assist with health behavior change. The “practice for change” construct is based on the praxis construct from Freire’s adult education model, which emphasizes active reflection and reflective behavior.^17,19^ “Practice for change” involves constantly deliberating behavior change, incorporating ongoing modifications to absolve ineffective strategies, addressing barriers, and staying focused on the health behavior change.^17^The “change in social environment” construct is developed from social support,^24^ helping relationships,^13^and environment,^19^ constructs. This construct involves establishing social support within the environment. Health educators may facilitate changes in the social environment, and this transformation may be natural or artificial.^17^

There is increasing evidence, including a meta-analysis, suggesting that public health and health promotion interventions that explicitly apply theoretical models from the social and behavioral sciences are more effective than interventions lacking a theoretical framework.^25^ Empirically testing theories/models is a critical step that should be conducted before utilizing them for intervention development.^26^ Therefore, the purpose of this study was to examine the utility of the MTM in predicting initiation and sustenance of PA behavior among college students. This was the first novel study to assess the predictive efficacy of the MTM in the health behavioral research domain. This study is useful in providing theoretical evidence to inform development of suitable PA-related interventions for college students.

## Materials and Methods

### Study design

In the current study, a cross-sectional design was utilized. Sample size was calculated using G* Power.^27^ An alpha of 0.05, power of 0.80, number of predictors as six (three for constructs in each model and three for control variables) and an effect size of 0.10 yielded a sample size of 143. A questionnaire was administered in October 2015 electronically to students enrolled in fall semester of 2015 at a large University in Southern, United States. All students received a link to the online questionnaire. Data were collected during a minimum three-week time period, and students received two reminder emails in the second and third week. Participants included in this study were over 18 years of age; did not have any medical condition that prevented them from being physically active; and did not engage in more than 150 minutes of moderate to vigorous intensity aerobic PA during the past week. The last criterion was important from the point of view of the MTM because this theory is about health behavior change. Informed consent was obtained electronically from all individual participants included in the study.

### Participants characteristics

Of 495 respondents, 190 met the inclusion criteria that comprised sedentary students. Among the included participants, 141 completed the survey. The majority of participants were females (72.3%) and Caucasians (70.9%). The mean (SD) age of the sample was 24.56 (8.19) years. One-third (33.3%) of the study sample were graduate students. Over half (54.6%) of the participants indicated their grade point average between 3.50 and 4.00 on a 4.00 scale. From all students, 75.9% reported living off-campus and 56.7% were currently working. Table 1 shows socio-demographic characteristics of the participants.

**Table 1:** Socio-demographic characteristics of the participants (n = 141)

	**Summary statistics** ^a^
Age (years)	24.56 (8.19)
Gender	
Male	39 (27.7%)
Female	102 (72.3%)
Race/Ethnicity	
White/Caucasian	100 (70.9%)
African American	24 (17.0%)
American Indian	8 (5.7%)
Hispanic American	4 (2.8%)
Other	5 (3.5%)
Class level	
Freshmen	18 (12.8%)
Sophomore	17 (12.1%)
Junior	25 (17.7%)
Senior	34 (24.1%)
Graduate	47 (33.3 %)
Current overall GPA	
Less than 1.99	2 (1.4%)
2.00–2.49	13 (9.2%)
2.50–2.99	19 (13.5%)
3.00–3.49	30 (21.3%)
3.50–4.00	77 (54.6%)
Living arrangements	
On campus	34 (24.1%)
Off-campus	107 (75.9%)
Work status	
Yes	80 (56.7%)
No	61 (43.3%)

^a^Mean (SD) is presented for age and n (%) for other variables.

### Instrumentation

A 37-item PA questionnaire was designed using relevant literature on PA and health behaviour research. Seven questions were about socio-demographic information: gender, age, ethnicity, class level, current grade point average, location of living, and work status. The remaining 30 items of the questionnaire assessed constructs of MTM.

### Initiation model

Five survey items assessed the advantages component of participatory dialogue. For example, “If you engage in more than 150 minutes of moderate to vigorous intensity aerobic PA every week you will be healthy.” Each item response ranged from never (=0) to always (=4). The scores for each item were added to achieve a total possible score for advantages (ranging from 0 to 20).

Five survey items assessed the disadvantages component of participatory dialogue. For example, “If you participate in more than 150 minutes of moderate to vigorous intensity aerobic PA every week you will be tired.” Each item response ranged from never (=0) to always (=4). The scores for each item were added to achieve a total possible score for disadvantages (ranging from 0 to 20). The score of disadvantages was subtracted from the score of advantages to obtain the score on participatory dialogue. It was hypothesized that the higher this score the greater was the likelihood of initiation of PA behavior.

Five survey items assessed behavioral confidence. For example, “How sure are you that you will be aerobically physically active with moderate to vigorous intensity for 150 minutes this week?” Each item response ranged from not at all sure (=0) to completely sure (=4). The scores for each item were added to achieve a total possible score for behavioral confidence (ranging from 0 to 20).

Three survey items assessed changes in physical environment. For example, “How sure are you that you will have a place to be aerobically physically active for 150 minutes per week?” Each item response ranged from not at all sure (=0) to completely sure (=4). The scores for each item were added to achieve a total possible score for physical environment (ranging from 0 to 12).

To assess initiation, participants were asked “How likely is it that you will increase your aerobic PA to 150 minutes in the upcoming weeks?” Response options ranged from not at all likely (=0) to completely likely (=4).

### Sustenance model

Three survey items assessed emotional transformation. For example, “How sure are you that you can direct your emotions/feelings to the goal of being aerobically physically active for 150 minutes every week?” Each item response ranged from not at all sure (=0) to completely sure (=4). The scores for each item were added to achieve a total possible score for emotional transformation (ranging from 0 to 12).

Three survey items assessed practice for change. For example, “How sure are you that you can keep a self-diary to monitor total time of your aerobic PA every week?” Each item response ranged from not at all sure (=0) to completely sure (=4). The scores for each item were added to achieve a total possible score for practice for change (ranging from 0 to 12).

Two survey items assessed changes in changes in social environment. For example, “How sure are you that you can get the help of a family member to be aerobically physically active for 150 minutes every week?” Each item response ranged from not at all sure (=0) to completely sure (=4). The scores for each item were added to achieve a total possible score for changes in social environment (ranging from 0 to 8).

To assess sustenance, participants were asked “How likely is it that you will increase your aerobic PA to150 minutes every week from now on?” Response options ranged from not at all likely (=0) to completely likely (=4).

### Face and content validity 

A panel of experts (n=6) in the area of health behavior research were invited to establish face and content validity of the questionnaire over a two round process. Two of the panel members were experts in PA, and three of the panel members were experts with college students. All of the panel members were experts with one or more theories/models in health education and in instrument development. The independent experts were asked to judge readability, relevance and clarity of the items. Based on the experts’ comments, minor alterations were made in the wording of the items. No items were removed from the questionnaire. The experts were unanimous about the adequacy of the content and face validity for each of the MTM subscales. The Flesch Kincaid Reading Ease of the instrument was 47.4 and Flesch-Kincaid Grade level of the instrument was 8.5.

### Construct validity

To assess the factor structure, we conducted a confirmatory factor analysis (CFA) in which we analyzed covariance matrices applying maximum-likelihood estimation using Mplus version 7.^28^ We used four indices to assess how well our models fit the data^29,30^: chi-square (χ^2^), root mean square error of approximation (RMSEA), comparative fit index (CFI), and standardized root mean square residual (SRMR). RMSEA values of 0.06 or less, in conjunction with CFI values of 0.95 or greater were considered indicative of good fit.^29^ Models were considered to have adequate fit if they met the less stringent, but traditionally accepted, values of 0.90 or greater for CFI, and values less than 0.08 for RMSEA. We also included SRMR because it has been identified as the index that is most sensitive to miss-specified factor covariances or latent structures.^29^ For SRMR, values less than 0.10 are acceptable, with values less than 0.08 being preferred.

### Reliability

For instrument’s reliability, internal consistency was determined with Cronbach alpha. An alpha coefficient greater than 0.60 was considered acceptable for subscales, as is recommended for measurement scales, especially in the case of new scales.^31^ Cronbach alpha coefficient of the subscales and the scale as a whole are depicted in Table 2. All the values were over 0.60 and thus acceptable.^31^

**Table 2:** Descriptive statistics of study variables (n=141)

**Constructs**	**Possible range**	**Observed range**	**Mean (SD)**	**Cronbach alpha**
Initiation	0-4	0-4	1.59 (1.18)	-
Participatory dialogue: advantages	0-20	0-20	14.56 (3.53)	0.87
Participatory dialogue: disadvantages	0-20	0-17	8.59 (2.98)	0.65
Participatory dialogue: advantages - disadvantages score	-20–+20	-10–+20	5.97 (5.23)	-
Behavioral confidence	0-20	0-12	6.52 (4.91)	0.83
Changes in physical environment	0-12	0-12	7.32 (4.03)	0.92
All constructs of initiation model	-	-	-	0.72
Sustenance	0-4	0-4	1.39 (1.17)	-
Emotional transformation	0-12	0-12	5.11 (3.06)	0.88
Practice for change	0-12	0-11	3.67 (2.80)	0.73
Changes in social environment	0-8	0-8	2.88 (2.11)	0.63
All constructs of sustenance model	-	-	-	0.84
Entire scale	-	-	-	0.83

### Data analyses

Descriptive statistical analyses were conducted to describe the study variables. Using stepwise multiple regression, best possible predictors of PA behavior change (i.e., initiation and sustenance) were assessed while controlling for demographic variables. For stepwise multiple regression the *apriori* criteria of probability of F to enter the predictor in the model was chosen as less than or equal to 0.05 and for removing the predictor as greater than or equal to 0.10. All data analyses were performed using IBM SPSS (version 20.0).

## Results

**Figure 1:**
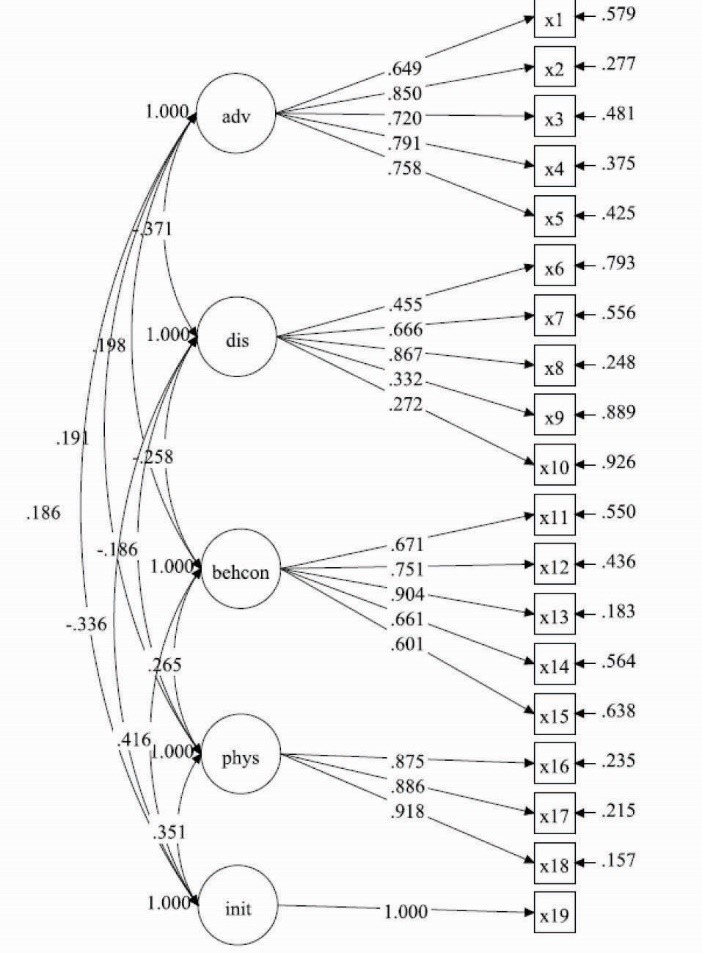
Confirmatory factor analysis (CFA) for initiation model.
Abbreviations: adv = Advantages; dis = Disadvantages; behcon = Behavioral confidence; phys = Changes in physical environment; init = initiation. All item loadings are significant to *P*<0.001.

A total of 143 participants provided complete data to examine construct validity and reliability of the instrument. While not ideal, previous Monte Carlo studies suggest that this sample size was sufficiently powered to evaluate the hypothesized measurement models.^32^ The path diagram in Figure 1 depicts the results for the CFA in Model 1. Fit for the model was good: χ^2^ = 253.92 (*df* = 143), *P*<0.001, χ^2^/df=1.78, CFI = 0.91, RMSEA = 0.07 (90% CI: 0.06-0.09), SRMR = 0.07. Additionally, all item loadings were significant at *P*<0.001. Latent covariances ranged from −0.37 between advantages and disadvantages, to 0.42 between initiation and disadvantages. Chi-square difference tests showed that an alternative one-factor model achieved poorer fit (χ^2^ = 937.94 [*df*= 152], *P*<0.001, CFI = 0.34, RMSEA = 0.19, SRMR = 0.17).

**Figure 2:**
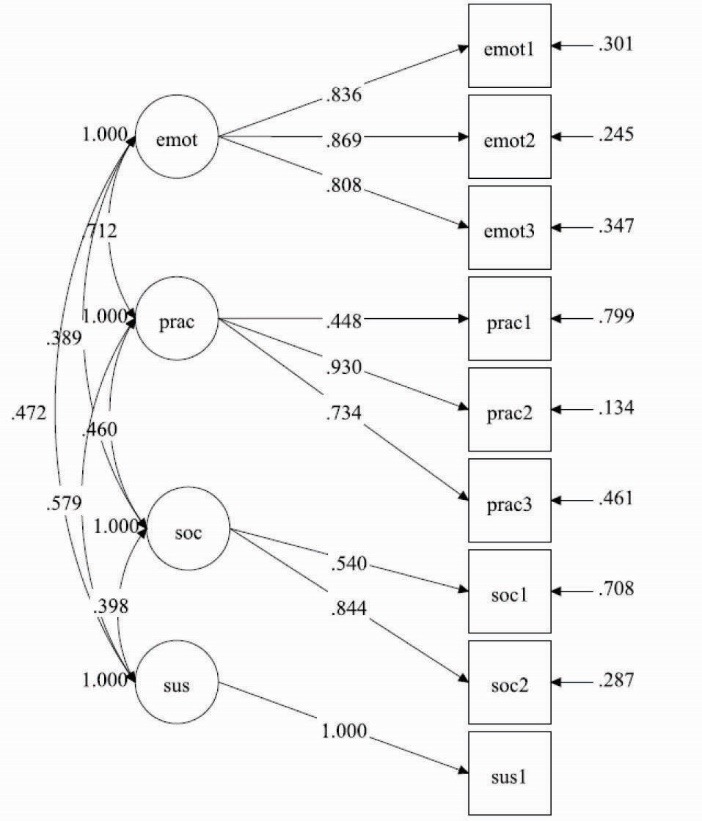
Confirmatory factor analysis (CFA) for sustenance model.
Abbreviations: emot = Emotional transformation; prac = Practice for Change; soc = Changes in Social Environment; sus = Sustenance. All item loadings are significant to *P*<0.001.

The path diagram in Figure 2 depicts the results for the CFA in Model 2. Fit for the model was good: χ^2^ = 19.40 (*df* = 22), *P*<0.001, χ^2^/df=0.88, CFI = 1.00, RMSEA = 0.00 (90% CI: 0.00-0.06), SRMR = 0.03. Additionally, all item loadings were significant at *P*<0.001. Latent covariances ranged from 0.39 between practice for change and sustenance, to 0.71 between emotional transformation and sustenance. Chi-square difference tests showed that an alternative one-factor model achieved poorer fit (χ^2^ = 112.10 [*df* = 27], *P*<0.001, χ^2^/df=4.15, CFI = 0.83, RMSEA = 0.15 [90% CI: 0.12-0.17], SRMR = 0.08). In sum, the analyses for both models support the hypothesized factor structure of the variables.

Table 2 depicts the descriptive statistics of study variables. For the construct of advantages, the mean was 14.39 (SD: 3.69) which shows participants’ attitude toward engagement in PA as moderately beneficial. The mean of 8.56 (SD: 3.03) for construct disadvantages indicates that participants sometimes view engagement in PA as disadvantageous. The mean score for behavioral confidence was 6.52 (SD: 4.91) which demonstrates that participants were less sure to do PA. The participants have a mean of **7**.32 (SD: 4.03) for the changes in physical environment which represents that participants were moderately sure to make changes in physical environment to be aerobically physically active. The mean score for initiation behavior was 1.59 (SD: 1.18, median 2, range 0-4) which demonstrated that participants were less likely to increase their aerobic PA to 150 minutes in the upcoming weeks.

The mean score for emotional transformation was 5.11(SD: 3.06) which demonstrated that participants were moderately sure in converting their emotions toward engagement in PA. The participants had a mean of 3.67 (SD: 2.80) for the practice of change which showed that they were less sure to prepare themselves to be physically active. The mean of 2.88 (SD: 2.11) which was on the lower end for the construct of “changes in social environment,” indicated that participants were less likely to take help of family member or friend to be physically active. For the sustenance behavior, the mean was 1.39 (SD: 1.17, median 1, range 0-4) which represents that participants were less likely to increase their aerobic PA to 150 minutes every week from now on. 

Results of stepwise multiple regression analysis for initiation model are depicted in Table 3. It showed that 26% of the variance in the initiation of PA was explained by advantages outweighing disadvantages, behavioral confidence, work status, and changes in physical environment, F (4, 135) = 13.220, *P*<0.001. For sustenance of PA model are depicted in Table 4. About 29.7% of the variance in the sustenance of PA was explained by emotional transformation, practice for change, and changes in social environment, F (3, 136) = 20.596, *P*<0.001.

**Table 3:** Parameter estimates based on stepwise regression analysis to predict initiation of physical activity behavior change (n=141)

**Variables**	**B**	**SE**_B_	**β**	**95% CI of B**	***P *** **value**
Advantages outweighing disadvantages	0.042	0.018	0.182	0.007–0.077	0.018
Behavioral confidence	0.075	0.019	0.310	0.038–0.112	<0.001
Changes in physical environment	2.062	0.023	0.208	0.016–0.107	0.008
Work Status	-0.509	0.175	-0.212	-0.855– -0.162	0.004

F (4, 135) = 13.220, *P*<0.001, R^2^(Adjusted R^2^) = 0.281 (0.260). Dependent variable is initiation of physical activity behavior change; B = unstandardized coefficient; SE_B_= standard error of the coefficient; β = standardized coefficient; *P* = level of significance.

**Table 4:** Parameter estimates based on stepwise regression analysis to predict sustenance of physical activity behavior change (n=141)

**Variables**	**B**	**SE**_B_	**β**	**95% CI of B**	***P*** ** value**
Emotional transformation	0.079	0.033	0.204	0.013–0.145	0.019
Practice for change	0.139	0.037	0.331	0.066–0.211	<0.001
Changes in social environment	0.098	0.042	0.175	0.014–0.181	0.022

F(3, 136) = 20.596, *P*<0.001, R^2^(Adjusted R^2^) = 0.312 (0.297) Dependent variable is sustenance of physical activity behavior change; B = unstandardized coefficient; SE_B_= standard error of the coefficient; β = standardized coefficient; *P*= level of significance.

## Discussion

The purpose of this study was to model PA behavior in college students using the constructs of MTM of health behavior change. The models were divided into initiation of behavior change and sustenance of behavior change. The salient conclusion from this empirical testing was that all the constructs proposed for initiation and all the constructs proposed for sustenance by MTM were found to be predictive of PA behavior in this sample of college students. For initiation of PA behavior, the constructs of advantages outweighing disadvantages, behavioral confidence, and changes in physical environment along with work status (*P*<0.001) predicted 26% of the variance which is substantially high for behavioral studies. Work status is also related to physical environment and can be construed as its component. The construct of advantages outweighing disadvantages has also been found to be significant as decisional balance in several TTM studies.^33,34^ The construct of behavioral confidence has substantial support from the work on self-efficacy,^35,36^ and perceived behavioral control.^37,38^ The present study underscored the importance of behavioral confidence in predicting starting of PA behavior. Finally, for initiation, physical environment including work status was found to be significant which also has support from the literature.^8,39^

For sustenance of PA behavior, the constructs of emotional transformation, practice for change, and changes in social environment (*P*<0.001) predicted 29.7% of the variance which is also fairly high for health behavior studies. The first construct of emotional transformation derived from emotional intelligence is relatively new in health behavior research and has not been explored with regard to PA behavior in college students.^23^ However, the present study lends credence to its application for PA promotion interventions. Likewise, the construct of practice for change derived from Freirian praxis has also not been operationalized in its entirety with regard to PA behavior in college students.^19^ However, some components like keeping a diary have been found to be effective in previous studies.^40^ The construct of social environment has also been found to be significant as helping relationships in a TTM study by Dishman et al^41^ or as social support.^38^ The role of family and friends in sustaining the behavior of PA in college students needs to be underscored.

So we see in this study that two parsimonious models with three constructs in each were able to account for a substantial proportion of variance in PA behavior in college students. The results from this empirical investigation are encouraging for designing PA promotion interventions in this high risk population. Regression results also show that the constructs do not seem to have much shared variance and hence the constructs are more or less independent of each other and are mutually exclusive lending support to this new theory for application to other health behaviors.

There were a total of 495 respondents in this study of which 190 (38.4%) met the inclusion criteria or were not getting enough PA. In other words, roughly 62% were meeting the goal of 150 minutes of PA per week which when compared to national data of 46% is encouraging and in line with the target set forth in Healthy People 2020.^2^ However, 38% sedentary students found in this study constitute a substantial number of students and more programming with regard to PA on University campuses need to be undertaken.

One of the finding in this study was that about 56% of the respondents worked with work status being negatively associated with intent to initiate PA. Working can be considered as part of the physical environment where by supportive environment and policies at work must nurture PA. Because of the competing demands of work on time, especially for college students who are in addition studying, college students often do not find enough time to balance work and PA which is a big barrier for PA.

If we closely look at the distribution of the scores for the constructs and behaviors, we find that they were on the lower end of the possible ranges thereby implying that there is lot of scope for improving these scores by interventions. In the section on implications for practice we have discussed specifically how these interventions can be planned based on MTM.

### Limitations 

This study was not without shortcomings. First, the study utilized a cross sectional study design which looks at all the variables at one time thereby nothing can be said about the temporal association of variables. Or in other words strictly speaking we cannot say that the constructs come before the behavior. However, previous theories have indicated that the attitudinal and environmental constructs precede the behavior so we can also assume the same for PA behavior in college students. Future studies can look at more robust study designs. Second, the actual behavior has not been measured by this study but a proxy intention for initiation and sustenance of behavior has been used in measurement which is subject to criticism. However, there is evidence in previous theories, particularly theory of reasoned action and theory of planned behavior^42^ that intentions precede behavior. So the measurement of behavior the way it has been done in this study can be justified. Future studies should look at measuring behavior more objectively. Third, the instrument was all self-report and that too introduces measurement bias. Self-reports are prone to dishonesty, false reporting under reporting or extreme reporting and such biases. However, when it comes to attitudinal assessments there are no other choices, so this limitation must be considered in that context. Finally, the test-retest (stability) reliability of the instrument was not conducted. Future studies replicating this study or working with other behaviors must also include test-retest reliability assessment.

### Implications for practice

It is evident from this study that there is a need to design PA promotion interventions for college students. The interventions can consist of one-on-one counseling, group interventions or campus wide campaigns. In order to influence initiation of PA behavior the first construct that needs to be modified is participatory dialogue in which the facilitator (health educator, health education specialist, faculty member, physician and so on) undertakes a dialogue with the individual, group or campus as a whole to underscore the advantages of PA behavior changeover disadvantages. At the same time, he or she builds behavioral confidence by delineating the PA behavior change into small steps, building confidence to perform the behavior in future, and strengthening the self. This can be done at the individual level by counseling and the group level by group discussion or other affective methods such as role play. At the campus level techniques such as psychodrama can be used. Finally, for altering physical environment a place should be available, affordable, and accessible for performing PA. The learners should also be well versed with all equipment required to be used.

In order to influence sustenance of PA behavior the first construct that needs to be modified is emotional transformation. The participants should be taught to direct their emotions such as anger, frustration, anxiety etc. into goal of performing 150 minutes of aerobic PA every week. Ability to constantly self-motivate oneself and overcome self-doubt into accomplishing this goal must also be taught. This can be done through one-on-one counseling or group discussion or for campus wide campaigns in the form of contests or involvement of social media. The second construct that needs modification is practice for change. This can be altered by keeping a diary, anticipating and overcoming barriers, having flexibility with plans. In other words, the participants must be encouraged to constantly reflect on their behavior change and maintain awareness. Finally, for influencing social environment help from family and friends must be mobilized for all three levels of interventions.

## Ethical approval

This study was approved by University of Mississippi Institutional Review Board (IRB). All procedures performed in studies involving human participants were in accordance with the ethical standards of the institutional and/or national research committee and with the 1964 Helsinki declaration and its later amendments or comparable ethical standards.

## Competing interests

None to declare.

## Authors contributions

Manuscript conceptualization: VKN and MS; Manuscript writing: VKN, MS, HPC, MJI, PJ, and MAF; Literature review: HPC and MJI; Instrument development: MS; Data collection: VKN and MAF; Data analysis: VKN, MS, and PJ; Data interpretation: VKN, MS, HPC, MJI, PJ, and MAF.
